# Developmental patterning of the sub-epidermal integument cell layer in *Arabidopsis* seeds

**DOI:** 10.1242/dev.146274

**Published:** 2017-04-15

**Authors:** Olivier Coen, Elisa Fiume, Wenjia Xu, Delphine De Vos, Jing Lu, Christine Pechoux, Loïc Lepiniec, Enrico Magnani

**Affiliations:** 1 Institut Jean-Pierre Bourgin, INRA, AgroParisTech, CNRS, University of Paris-Saclay, Route de St-Cyr (RD10), Versailles Cedex 78026, France; 2 Ecole Doctorale 567 Sciences du Végétal, University Paris-Sud, University of Paris-Saclay, bat 360, Orsay Cedex 91405, France; 3 INRA, Génétique Animale et Biologie Intégrative, Domaine de Vilvert, Jouy-en-Josas Cedex 78352, France

**Keywords:** Fertilization, Seed coat, Integuments, Tissue cross-talk, Seed development

## Abstract

Angiosperm seed development is a paradigm of tissue cross-talk. Proper seed formation requires spatial and temporal coordination of the fertilization products – embryo and endosperm – and the surrounding seed coat maternal tissue. In early *Arabidopsis* seed development, all seed integuments were thought to respond homogenously to endosperm growth. Here, we show that the sub-epidermal integument cell layer has a unique developmental program. We characterized the cell patterning of the sub-epidermal integument cell layer, which initiates a previously uncharacterized extra cell layer, and identified TRANSPARENT TESTA 16 and SEEDSTICK MADS box transcription factors as master regulators of its polar development and cell architecture. Our data indicate that the differentiation of the sub-epidermal integument cell layer is insensitive to endosperm growth alone and to the repressive mechanism established by FERTILIZATION INDEPENDENT ENDOSPERM and MULTICOPY SUPPRESSOR OF IRA1 Polycomb group proteins. This work demonstrates the different responses of epidermal and sub-epidermal integument cell layers to fertilization.

## Abstract (precis)

**Summary:** The sub-epidermal integument cell layer of the *Arabidopsis* seed coat is insensitive to endosperm growth alone and displays a unique response to fertilization.

## INTRODUCTION

In angiosperms, seed development starts with the double fertilization of the egg and central cell in the ovule that leads to the formation of embryo and endosperm, respectively. Proper seed formation is then achieved through tight spatial and temporal coordination of embryo, endosperm, and seed maternal tissues (Ingram, 2010).

In *Arabidopsis*, ovule primordia are composed of three functional domains: the funiculus, which transports nutrients from the mother plant; the chalaza, which initiates two integuments; and the nucellus, which gives rise to the female gametophyte. Both inner (ii) and outer (oi) integuments are composed of two epidermal cell layers (ii1, ii2, oi1 and oi2) which grow by anticlinal cell divisions to progressively surround the female gametophyte. At the end of ovule development, the ii1 undergoes periclinal cell divisions to give rise to a sub-epidermal integument cell layer, the so-called ii1′ (Fig. S1) (Schneitz et al., 1995; Debeaujon et al., 2003). The fertilization-independent development of the ovule is repressed by a class of Polycomb group (PcG) proteins named FERTILIZATION INDEPENDENT SEED (FIS). In particular, the FERTILIZATION INDEPENDENT ENDOSPERM (FIE) and MULTICOPY SUPPRESSOR OF IRA1 (MSI1) FIS PcG proteins act sporophytically to repress the differentiation of the integuments (Roszak and Köhler, 2011). After fertilization of the central cell, the endosperm initiates a signal, through the action of the MADS box transcription factor AGAMOUS-LIKE 62, that relieves the FIS-mediated repression and leads to the differentiation of the ovule integuments into seed coat (Roszak and Köhler, 2011). Results from Figueiredo et al. (2016) suggest auxin as the putative fertilization signal that coordinates endosperm and seed coat development. Nevertheless, exclusive fertilization of the egg cell by *kokopelli* or *cyclin dependent kinase a;1* mutant pollen triggers partial differentiation of the ovule integuments surrounding the female gametophyte (Ungru et al., 2008; Kasahara et al., 2016). Partial differentiation of the ovule integuments is also initiated by the pollen tube content of the *generative cell-specific 1* (*gcs1*) mutant, whose sperm cells fail to fertilize the female gametophyte (Kasahara et al., 2016). Furthermore, *gcs1* pollen tube content induces full differentiation of the integuments of *medea* FIS PcG mutant ovules, which undergo fertilization-independent proliferation of the central cell. These data suggest that pollen tube rupture and not fertilization enables the central cell to initiate the signaling pathway that leads to differentiation of the seed coat. In response to the endosperm signal, the five seed coat cell layers undergo a rapid phase of cell division and expansion, and follow different cell fates (Haughn and Chaudhury, 2005). The MADS box protein TRANSPARENT TESTA 16 (TT16) works downstream of FIE and MSI1 to promote the production of proanthocyanidins (PAs) in the ii1, the innermost seed coat cell layer (also known as the endothelium) (Xu et al., 2016). Finally, endosperm and seed coat coordinate their growth through a cross-talk signaling pathway (Ingram, 2010) that was first identified in the study of the maternally acting *TRANSPARENT TESTA GLABRA 2* (*TTG2*) and zygotically acting *HAIKU* (*IKU*) genes. Both *ttg2* and *iku* mutants show premature arrest of endosperm development and reduced seed size, indicating that the developmental interaction between seed coat and endosperm orchestrates early seed growth with limited embryo contribution (Garcia et al., 2005).

To date, all seed integuments were thought to respond to the same signaling pathway initiated by endosperm growth. Here, we show that the sub-epidermal ii1′ has a unique developmental program. We followed ii1′ cell patterning from its inception in the ovule until its differentiation in the seed. We demonstrated that the ovule ii1′ undergoes periclinal cell divisions to originate a previously unnoted sixth integument cell layer. We characterized the redundant role of the MADS box transcription factors TT16 and SEEDSTICK in promoting ii1′ formation in the ovule. Furthermore, we showed that TT16 regulates the proximal-distal patterning of the ii1′, preventing its development in the micropylar zone. After fertilization, TT16 is implicated in cell orientation and differentiation of the ii1′. Our analyses indicate that ii1′ growth does not respond to the FIE and MSI1 PcG repressive mechanism and to endosperm growth alone, which regulate development of the epidermal integument cell layers. These data suggest that epidermal and non-epidermal integument cell layers respond to different fertilization signaling pathways.

## RESULTS

### The ii1′ gives rise to a sixth integument cell layer

**Fig. 1.**
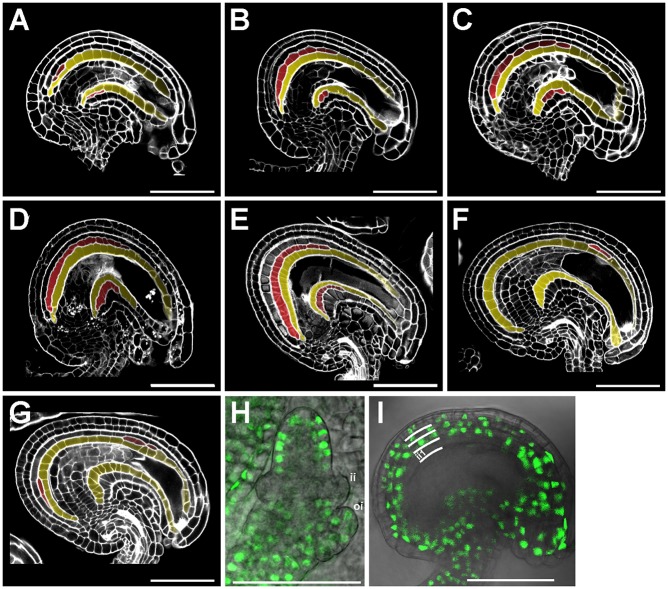
**TT16 and STK promote ii1 periclinal cell divisions.** (A-C) Central longitudinal sections of wild-type ovules progressing (from A to C) through stage 3-VI imaged using the mPS-PI technique. Ecotype Col. (D-G) Central longitudinal sections of wild-type (D), *stk* (E) and *stk;tt16* (F,G) ovules at stage 4-I imaged using the mPS-PI technique. Ecotype Col. (H,I) GFP fluorescence images superimposed on brightfield images of *ProSTK:gSTK-GFP* ovules at stage 2-III (H) and 3-V (I). Integument cell layers are marked by white lines. ii1 and ii1′ are highlighted in yellow and red, respectively. Scale bars: 50 µm.

**Fig. 2.**
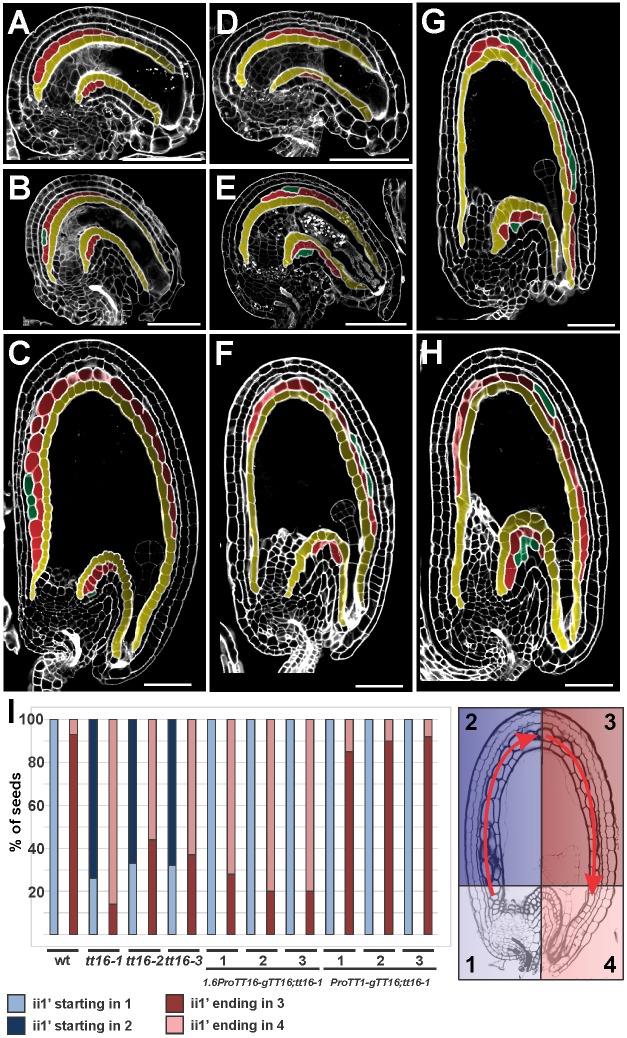
**TT16 regulates ii1′ proximal-distal developmental patterning.** (A-C) Central longitudinal sections of wild-type ovule at stage 3-VI (A) and seeds at 2 (B) and 4 (C) DAF imaged using the mPS-PI technique. Ecotype Ws. (D-H) Central longitudinal sections of *tt16* ovule at stage 3-VI (D) and seeds at 2 (E) and 4 (F-H) DAF imaged using the mPS-PI technique. Ecotype Ws. (I) In blue, percentage of seeds (6 DAF) with the ii1′ beginning in zone 1 (light blue) or zone 2 (dark blue). In red, percentage of seeds with the ii1′ ending in zone 3 (dark red) or zone 4 (light red). Seed zones 1-4 are schematized and color-coded on the right. Ecotype Ws. *n*=30. ii1, ii1′ and ii1″ are highlighted in yellow, red and green, respectively. Scale bars: 50 µm.

The last stage of *Arabidopsis* ovule development, stage 3-VI, is marked by the formation of the inner integument 1′ (ii1′) by periclinal cell divisions of the ii1, the innermost integument cell layer (Fig. S1) (Schneitz et al., 1995; Debeaujon et al., 2003). To thoroughly characterize the process of ii1′ formation we analyzed central longitudinal sections of *Arabidopsis* ovules at stage 3-VI, three-dimensionally reconstructed using the modified pseudo-Schiff propidium iodide imaging technique (mPS-PI, see Materials and Methods). Cells of the ii1 underwent periclinal cell divisions starting from the chalazal pole and progressed toward the micropyle region without interruptions (Fig. 1A-C, ii1 and ii1′ are highlighted in yellow and red, respectively, throughout). The first cell of the ii1, identified as the cell following the merging of ii1 and ii2 in the chalazal pigment strand (Fig. S1), did not undergo periclinal cell divisions (Fig. 1A-C). The ii1′ arose from the second, third or fourth cell of the ii1 and developed toward the micropyle without ever reaching it (Fig. 1A-C). At the end of stage 3-VI/beginning of stage 4-I, we observed additional periclinal cell division in the ii1′ that gave rise to a sixth integument cell layer, which we named ii1″ (Fig. 2B, ii1″ is highlighted in green throughout). The ii1″ was limited to the chalazal area, as fertilization followed rapidly and led to the differentiation of the integuments, but persisted after fertilization showing a developmental patterning similar to ii1′ (Fig. 2C). The ii1″phenotype was more penetrant in the Wassilewskija accession (70% of the ovules) compared with Columbia (35% of the ovules). Both ii1′ and ii1″ are sub-epidermal cell layers, unlike the other integument cell layers which are all L1 epidermal layers (Fig. 2B,C) (Debeaujon et al., 2003).

### TT16 and STK regulate ii1′ cellular patterning

**Fig. 3.**
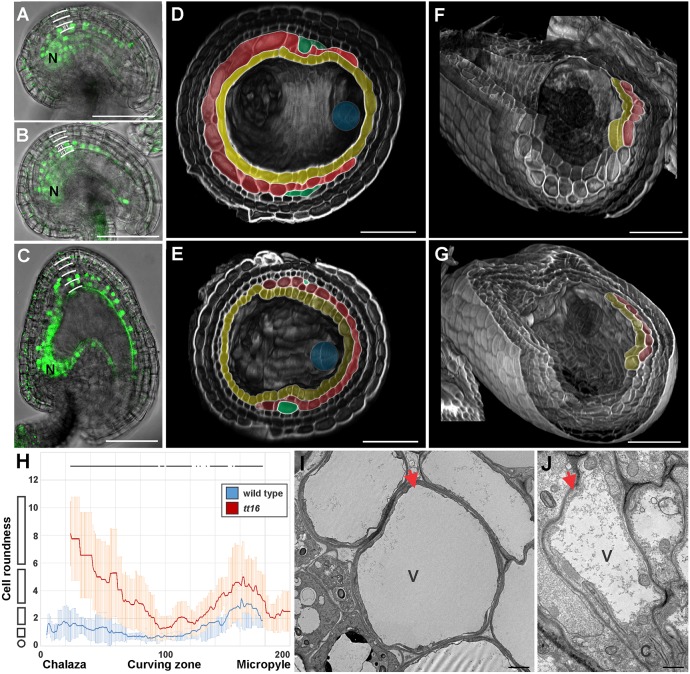
**TT16 regulates ii1′ cell orientation and differentiation.** (A-C) GFP fluorescence images superimposed on brightfield images of *3.4ProTT16:gTT16-GFP* ovules at stage 3-V (A) and 3-VI (B) and seed at 2 DAF (C). Integument cell layers are marked by white lines. N, nucellus. (D,E) Three-dimensional transverse sections of wild-type (D) and *tt16* (E) seeds at 4 DAF imaged using the mPS-PI technique. Embryos are highlighted in blue. Ecotype Ws. (F,G) Three-dimensional transverse and longitudinal sections of wild-type (F) and *tt16* (G) seeds at 4 DAF imaged using the mPS-PI technique. Ecotype Ws. (H) Average ii1′ cell roundness (see Materials and Methods) along the seed coat proximal-distal axis (arbitrarily divided in 201 points) as observed in central longitudinal sections of *tt16* (red) and wild-type (blue) seeds at 4 DAF. The shapes on the left of the graph exemplify how cell shape changes along the *y*-axis. Lines at the top of the graph indicate regions of statistically significant difference between wild type and *tt16* (two-tailed Student's *t*-test, *P*<0.05). Error bars indicate s.d. *n*=12. Ecotype Ws. (I,J) Transmission electron microscopy images of longitudinal sections of the curving zone of wild-type (I) and *tt16* (J) seed at 4 DAF. Red arrows indicate ii1′ cells. V, vacuole; C, cytoplasm. Ecotype Ws. Endothelium, ii1′ and ii1″ are highlighted in yellow, red and green, respectively. Scale bars: 50 µm in A-G; 1 µm in I,J.

The ovules of the *Arabidopsis transparent testa 16;seedstick* (*tt16;stk*) double mutant have been shown to carry only four integument cell layers and have been interpreted as missing the ii1 (Mizzotti et al., 2012). The progression of wild-type integument development argues against such an interpretation, as ii1′ is the last integument cell layer to appear (Schneitz et al., 1995). We therefore analyzed the development of the *tt16;stk* innermost integument cell layer by mPS-PI in ovules and seeds. We detected few periclinal cell divisions occurring stochastically along the integument proximal-distal axis (Fig. 1F,G, Fig. S2) when compared with wild type (Fig. 1D, Fig. S2). In 89% of the *tt16;stk* samples we observed a patchy pattern of periclinal cell divisions (Fig. 1G, Fig. S2), a phenotype never observed in wild type (Fig. 1D, Fig. S2). These data strongly suggest that *tt16;stk* ovules are impaired in the periclinal cell divisions of the innermost integument cell layer that, in wild-type ovules, give rise to the ii1′. Only 9.5% of *tt16;stk* seeds displayed a few ii1″ cells. Although the *stk* single mutant exhibited a pronounced ovule shape defect (Mizzotti et al., 2014), it did not show any ii1′ or ii1″ aberrant phenotype (Fig. 1E, Fig. S2). However, 74% of *tt16* ovules displayed a more distal ii1′ and ii1″ (after the fourth ii1 chalazal cell) compared with wild type (Fig. 2D), and 16% of *tt16* ovules developed a long ii1″ that resulted in a true six-cell layered seed coat (Fig. 2G). In wild-type seeds, the ii1′ extended through the curving zone until approximately halfway towards the micropylar pole (Fig. 2C,I). As a consequence, transverse imaging of wild-type seeds beyond their midline did not show the ii1′ around the developing embryo (Fig. 3D). Conversely, in the majority of *tt16* seeds, the ii1′ was still present beyond the seed midline and entered the micropylar region (Fig. 2F-I). Transverse views of *tt16* seeds beyond their midline strikingly showed a shift of the ii1′ toward the side occupied by the developing embryo (Fig. 3E). Thus, *tt16* developing embryos were often compressed by the concomitant mechanical action of the seed ii1′ invading the micropylar pole (Fig. 2H) and the persistence of the nucellus at the chalazal pole (Xu et al., 2016). Embryo cell morphology and proliferation in the *tt16* mutant appeared comparable to wild type (Fig. 2C-H). The maternal origin of the compressed embryo phenotype was confirmed by analyzing *tt16* ovules fertilized with wild-type pollen (Fig. S3). This phenomenon could be partially responsible for the arrested seed phenotype that we observed in *tt16* mutant siliques. Six-week-old *tt16* plants carried 7-23% (Ws background) or 6-33% (Col background) arrested seeds per silique (*n*=20 siliques observed), whereas control wild-type plants exhibited a low rate (1.4-3.2% in Ws or 0-12% in Col) of seed arrest in the same growing conditions (*n*=13 siliques observed in Ws and *n*=20 in Col). Embryo arrest in *tt16* seeds might also be caused by defects in nutrient transport through the seed coat (Chen et al., 2015). Altogether, these data indicate that STK and TT16 redundantly promote ovule ii1′ formation, whereas TT16 alone regulates the positioning of ii1′ along the ii1 proximal-distal axis.

To test if *TT16* expression correlates with the development of the ovule ii1′, we created a marker line carrying the 3.4 kb *TT16* promoter region and genomic sequences translationally fused to green fluorescent protein (GFP). We have previously shown that this *TT16* genomic region fully complements *tt16* mutant phenotypes in the nucellus and endothelium (Xu et al., 2016). We detected fluorescence in the nuclei of the proximal region of the ovule ii1 at stage 3-V (Fig. 3A) and in the developing ii1′ (Fig. 3B) at stage 3-VI. GFP expression extended to more distal cells of the endothelium and ii1′ in seeds at 2 days after flowering (DAF; see Materials and Methods) but never reached the micropyle (Fig. 3C). The *TT16* expression pattern in the ovule ii1 marks in advance the development of ii1′ and might therefore be responsible for the correct positioning and progression of ii1 periclinal cell divisions. In one possible scenario, *TT16* might define only the position of the first ii1′ cell, whereas the following periclinal cell divisions continue by default until ovule maturity. Alternatively, *TT16* might define the precise ii1′ spatial window from beginning to end. To distinguish between these two hypotheses, we expressed *TT16* under the control of the 1.6 kb *TT16* promoter, which marks the first two or three proximal cells of the ovule ii1 and the nucellus, in a *tt16* mutant background (*1.6ProTT16:gTT16;tt16*) (Xu et al., 2016). Three independent transgenic lines fully complemented the *tt16* phenotype at the ii1′ chalazal end (Fig. 2I, Fig. S4). Nevertheless, they failed to complement growth of ii1′ into the micropylar area and produced seeds that were completely covered by five seed coat cell layers (Fig. 2I, Fig. S4). These data might highlight the limited extent of TT16 non-cell-autonomous effects (Xu et al., 2016) along the ii1 proximal-distal axis. Nevertheless, these same complementation lines fully restored the arrested production of PAs along the entire *tt16* endothelium (Xu et al., 2016). Therefore, our analyses strongly suggest that *TT16* marks the end of the ii1 periclinal cell divisions in a cell-autonomous fashion, in contrast to the non-cell-autonomous regulation of PA biosynthesis. In line with this hypothesis, early expression of *TT16* in the ii1, with the exception of the micropylar zone, under control of the *TT1* promoter region (Fig. S4) fully complemented the *tt16* ii1′ phenotype at the chalazal and micropylar regions (*ProTT1:gTT16;tt16*, Fig. 2I, Fig. S4). Embryos of *1.6ProTT16:gTT16;tt16* seeds, which displayed a nucellus of wild-type appearance (Xu et al., 2016) and a five cell layered seed coat at the micropylar zone, were never compressed by the surrounding seed coat (Fig. S4) as observed in *tt16* seeds (Fig. 2H, Fig. S3). We could not analyze seeds carrying a seed coat of wild-type appearance and persistent nucellus, as the nucellus of *ProTT1:gTT16;tt16* seeds degenerates due to TT16 non-cell-autonomous effects (Xu et al., 2016). The negative effect of ii1′ growth around the embryo combined with nucellus persistence might have driven the evolution of a tight regulatory mechanism that coordinates ii1′ and nucellus development.

The *STK* promoter region and genomic sequences drove *GFP* expression in the ovule outer integument (oi) and ii2 throughout ovule development (Fig. 1H,I) (Mizzotti et al., 2014). The absence of *STK* expression in the ii1 suggests that STK affects ii1 periclinal cell divisions non-cell-autonomously.

### TT16 regulates ii1′ cell architecture

In line with previous analyses (Nesi et al., 2002), longitudinal sections of *tt16* seeds showed thinner and more elongated cells in the endothelium and ii1′ when compared with wild type (Fig. 2F-H). To better characterize such morphological anomalies, we analyzed images of three-dimensionally reconstructed seeds using the mPS-PI technique. In the proximal half of the wild-type seed coat, endothelium and ii1′ cells tended to be tubular in shape and oriented perpendicularly to the longitudinal axis of the seed (Fig. 3F). Thus, they appeared round in longitudinal section (Fig. 2C) and elongated in transverse section (Fig. 3D). In the *tt16* mutant, endothelium and ii1′ cells appeared equally tubular but aligned along the proximal-distal axis of the seed, and thus perpendicular to wild-type cells (Fig. 3G). We noticed that the orientation of these cells changed along the proximal-distal axis in both wild-type and *tt16* seeds. Longitudinal sections of wild-type ii1′ displayed cells that were more elongated in the chalazal and micropylar zones compared with the curving zone (Fig. 3H, Fig. S5). The *tt16* ii1′ followed the same trend of cell elongation along the proximal-distal axis but showed cells that were strikingly more elongated than in the wild type (Fig. 3H). These analyses clearly suggest that TT16 regulates endothelium and ii1′ cell architecture, and this probably underlies the misshapen *tt16* seed phenotype (Nesi et al., 2002). TT16 might establish ii1 and ii1′ cell orientation in the ovule but its mutant phenotype became evident in seeds after cell elongation. Such a phenotype accentuates the proximal-distal positional defect of the *tt16* ovule ii1′ described above.

The ii1′ undergoes a drastic cell expansion in response to fertilization (Fig. 2C, Fig. 3D) (Beeckman et al., 2000). Transmission electron microscopy imaging revealed highly vacuolated ii1′cells (Fig. 3I) at the curving zone of wild-type seeds. In *tt16* seeds, these cells appeared more cytoplasmic, suggesting that their cell expansion might be impaired (Fig. 3J). Similarly, *tt16* seeds fail to correctly differentiate the endothelium as they do not produce PAs (Nesi et al., 2002). Altogether, these data indicate that TT16 regulates cell orientation and differentiation of the endothelium and seed ii1′.

### Unique response of ii1′ to fertilization

**Fig. 4.**
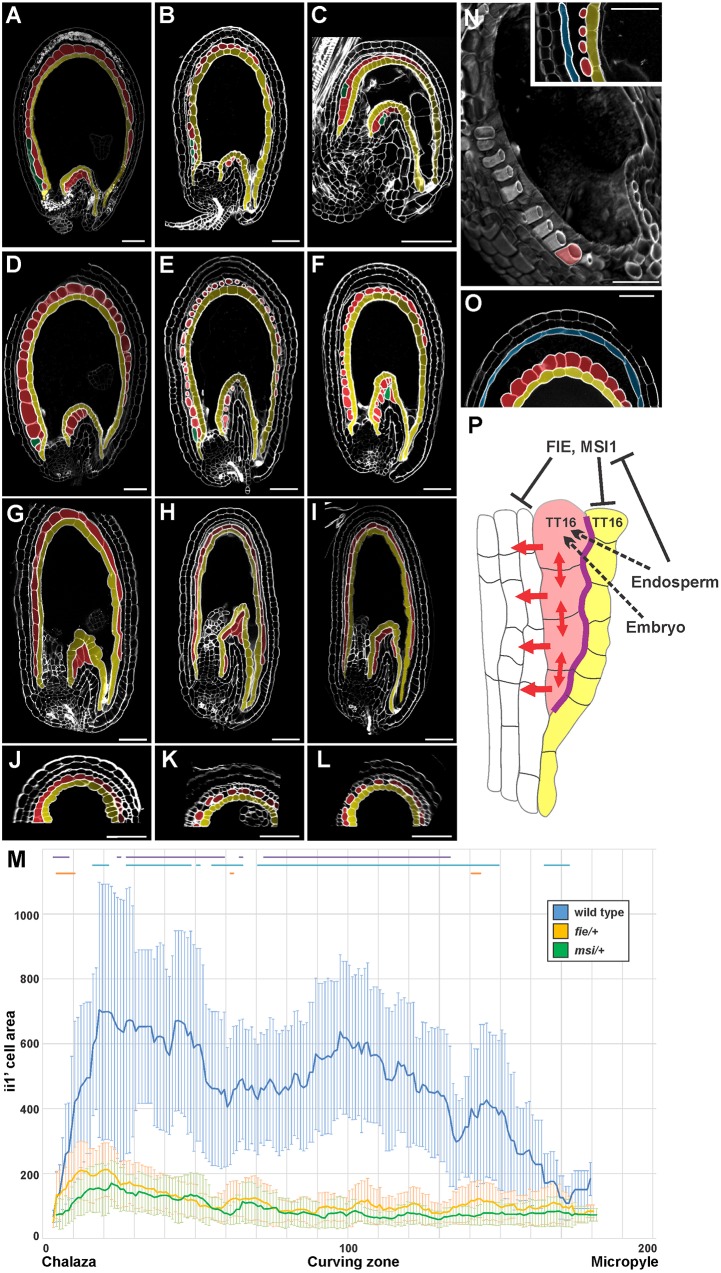
**Unique response of ii1′ to fertilization.** (A-C) Central longitudinal sections of wild-type (A), *kpl* endosperm-only (B) and *kpl* embryo-only (C) seeds at 6 DAF. Ecotype Ws. (D-F) Central longitudinal sections of wild-type (D), *fie/+* enlarged autonomous (E) and *msi1/+* enlarged autonomous (F) seeds at 6 DAF. Ecotype Col. (G-I) Central longitudinal sections of *tt16* (G), *tt16;fie/+* enlarged autonomous (H) and *tt16;msi1/+* enlarged autonomous (I) seeds at 6 DAF. Ecotype Col. (J-L) Transverse sections of *tt16* (J), *tt16;fie/+* enlarged autonomous (K) and *tt16;msi1/+* enlarged autonomous (L) seeds at 6 DAF. Ecotype Col. (M) Average ii1′ cell area (see Materials and Methods) along the seed coat proximal-distal axis (arbitrarily divided in 201 points) as observed in central longitudinal sections of wild-type seeds (blue), and *fie/+* (orange) and *msi1/+* (green) enlarged autonomous seeds at 6 DAF. Lines at the top of the graph indicate regions of statistically significant difference between wild type and *fie/+* (purple), wild type and *msi1/+* (blue), and *fie/+* and *msi1/+* (orange) (two-tailed Student's *t*-test, *P*<0.05). Error bars indicate s.d. *n*>12. Ecotype Col. (N) Three-dimensional longitudinal section of a *fie/+* enlarged autonomous seed at 6 DAF. Only one ii1′ cell is highlighted in red. The inset shows the central longitudinal section of the chalazal side of the same seed. Ecotype Col. (O) Central longitudinal section of the curving zone of a wild-type seed at 6 DAF, gently squeezed between slide and coverslip. Ecotype Col. (A-L,N,O) Imaged using the mPS-PI technique. (P) Model for the development of the seed ii1′. Black and red arrows indicate functional relationships and mechanical forces, respectively. Dashed lines indicate hypothetical relationships. The purple line indicates strong cell-cell adhesion. Endothelium, ii1′, ii1″ and ii2 are highlighted in yellow, red, green and light blue, respectively. Scale bars: 50 µm.

Fertilization of the central cell has been shown to trigger the differentiation of seed maternal tissues (Roszak and Köhler, 2011; Xu et al., 2016). To test the effect on ii1′ differentiation of each fertilization event independently, we examined the seed coat of the *kokopelli* (*kpl*) mutant, which displays random single-fertilization events (Ron et al., 2010). *kpl* seeds carrying only the embryo (*kpl* embryo-only seeds) have a small and partially differentiated seed coat (Roszak and Köhler, 2011; Kasahara et al., 2016). By contrast, *kpl* seeds that develop only the endosperm (*kpl* endosperm-only seeds) produce a large seed coat with a fully differentiated endothelium, as suggested by PA accumulation (Roszak and Köhler, 2011). In line with previous results, the ii1′ of *kpl* embryo-only seeds resembled that of undifferentiated ovules (Fig. 4C compared with Fig. S6). In *kpl* endosperm-only seeds, the ii1′ cells did not expand in coordination with the development of the other integument cell layers, creating empty spaces (Fig. 4B compared with Fig. 4A). Development of ii1′ was unaffected in *kpl* mutant ovules (Fig. S6). These data suggest that ii1′ differentiation requires fertilization of both the egg and central cell.

The *FIE* and *MSI1* PcG genes are expressed in all ovule integument cell layers (Köhler et al., 2003; Xu et al., 2016) and are thought to repress their fertilization-independent development (Roszak and Köhler, 2011). *fie* and *msi1* mutations are haploinsufficient, and unfertilized *fie/+* and *msi1/+* pistils carry a number of enlarged autonomous seeds that exhibit an apparently developed seed coat that accumulates PAs and a degenerated nucellus, both hallmarks of fertilization (Roszak and Köhler, 2011; Xu et al., 2016). However, we observed that *fie/+* and *msi1/+* enlarged autonomous seeds displayed an underdeveloped and discontinuous ii1′ made of unexpanded cells and large empty spaces (Fig. 4E,F compared with Fig. 4D; Fig. 4M). Furthermore, we found a higher number of ii1′ cells in *fie/+* and *msi1/+* enlarged autonomous seeds compared with wild-type seeds and ovules (Fig. S6). By contrast, ii1′ development was unaffected in *fie/+* and *msi1/+* developing ovules and seeds (Fig. S6). We speculate that the differentiation of the ovule ii1′ is not solely repressed by FIE and MSI1 and might require the action of other FIS PcG proteins or a different molecular mechanism. Alternatively, it might lack any repressive mechanism and respond to the positive stimulus of double fertilization. Altogether, these results demonstrate that epidermal and non-epidermal integument cell layers are regulated by different fertilization signaling pathways.

To test if TT16 plays a role in the process, we looked at *tt16;fie/+* and *tt16;msi1/+* enlarged autonomous seeds. We focused our attention on the fraction of seeds that did not display a strong *tt16* proximal-distal polarity defect in the ii1′ in order to better compare the results with single PcG mutants. The ii1′ of both double mutants resembled that of *tt16* fertilized seeds but with less expanded cells (Fig. 4H,I compared with Fig. 4G). Since *tt16* ii1′ cells are oriented perpendicularly to wild-type cells, we analyzed transverse sections of *tt16;fie/+* and *tt16;msi1/+* enlarged autonomous seeds. We observed that the ii1′ did not expand correctly and showed large empty spaces when compared with *tt16* fertilized seeds (Fig. 4K,L compared with Fig. 4J), a phenotype similar to that observed in *fie/+* and *msi1/+* longitudinal seed sections (Fig. 4E,F). These results suggest that TT16 does not affect ii1′ responsiveness to the fertilization signals and to the growth of the neighboring integument cell layers.

*fie/+* and *msi1/+* enlarged autonomous seeds showed cells of the ii1′ physically disconnected from one another and from the ii2 (Fig. 4N). We tested whether this is also the case in wild-type seeds by gently squeezing them in between slide and coverslip. In all our attempts (*n*=20), ii1′ was easily detached from ii2, whereas it always remained anchored to the endothelium (Fig. 4O). The only other tissues that were occasionally separated during these experiments were ii2 and oi1, which are separated by cutin-like material (Creff et al., 2015) that allows tissue sliding (Tsuwamoto et al., 2008). These results indicate that the seed ii1′ is loosely attached to the ii2 and suggest that it might develop unique cell wall properties.

## DISCUSSION

All *Arabidopsis* seed coat cell layers were thought to respond homogeneously to the fertilization of the central cell and to grow in a coordinated fashion with the endosperm. Our genetic analyses reveal the unique developmental program of the sub-epidermal integument cell layer. The underdeveloped ii1′ of *kpl* endosperm-only seeds and *fie/+* and *msi1/+* enlarged autonomous seeds contrasts with the growth of the other seed coat tissues and suggests a role for the embryo in early seed coat development (Fig. 4P). Furthermore, these data indicate a lack of developmental cross-talk between epidermal and sub-epidermal integument cell layers. In one scenario, the embryo might be necessary to establish developmental coordination between ii1′ and the other integument cell layers. Alternatively, the ii1′ might have evolved to grow independently of the other seed coat cell layers and arrest its development when compressed between the neighboring cell layers, solely under the constraints of mechanical forces. In line with the latter hypothesis, the seed coat of the *tt16* and *tt16;stk* mutants grows regardless of the displaced or absent ii1′. Furthermore, the higher number of ii1′ cells observed in *fie/+* and *msi1/+* enlarged autonomous seeds might be interpreted as a compensation for the lack of ii1′ cell expansion driven by the absence of mechanical constraints. Compared with the epidermal integument cell layers, the ii1′ originates by periclinal cell divisions. This process might underlie the unique properties of this tissue by leading to unequal partitioning of signaling components or a change in the epigenetic state.

The morphology of the ii1′, which is highly vacuolated and free to expand on the abaxial side, resembles that of the leaf parenchyma and suggests a role in cushioning seed coat development (Fig. 4P). We speculate that the ii1′ fine-tunes seed growth by offsetting perturbations in its developmental program. For example, the ii1′ might fill the empty space left by uneven growth of the seed inner and outer integuments or adjust seed coat development to the turgor pressure exerted by the endosperm (Beauzamy et al., 2016). This function might be better achieved through mechanical constraints than tight developmental control as it would provide a level of flexibility that is highly favorable to sessile organisms, which have to adapt to environmental changes.

## MATERIALS AND METHODS

### Plant materials

*Arabidopsis thaliana* plants of ecotype Columbia (Col) or Wassilewskija (Ws) were used as wild-type controls as appropriate. *kpl-1*, *tt16-2* and *tt16-3* lines are in the Ws accession (Nesi et al., 2002; Ron et al., 2010). *stk-2*, *stk-2;tt16-6*, *fie-12/+* and *msi1-1/+* lines are in the Col accession (Roszak and Köhler, 2011; Mizzotti et al., 2012). The *tt16-1* mutant was isolated in the Ws accession and then backcrossed to the Col accession more than three times (Nesi et al., 2002; Xu et al., 2016). Col and Ws *tt16-1* mutants were used as appropriate. Unless noted, *tt16* refers to *tt16-1*. The *tt16-1;fie-12/+* and *tt16-1;msi1-1/+* lines were generated in the Col accession (Xu et al., 2016).

Days after flowering (DAF) were counted starting from the emergence of the pistil from closed flowers (Xu et al., 2016). Both DAF and embryo development were used to determine seed developmental stages.

### Cloning

The 3.4 kb *TT16* promoter and genomic sequence was PCR amplified without stop codon using forward (5′-TCAATGGTAATTCATGAGGACGTTG-3′) and reverse (5′-ATCATTCTGGGCCGTTGGATCGTT-3′) primers that had the *attB1* (5′-GGGGACAAGTTTGTACAAAAAAGCAGGCT-3′) and *attB2* (5′-GGGGACCACTTTGTACAAGAAAGCTGGGTC-3′) GATEWAY recombination sites at the 5′-end, respectively. The PCR product was amplified by high-fidelity Phusion DNA polymerase (Thermo Fisher Scientific), recombined into the pDONR207 vector (BP Gateway reaction) according to the manufacturer's instructions (Thermo Fisher Scientific), and sequenced. The PCR product cloned into the DONR vector was then recombined into the pMDC107 binary vector (Curtis and Grossniklaus, 2003) (LR Gateway reaction) according to the manufacturer's instructions (Thermo Fisher Scientific). *1.6ProTT16:gTT16;tt16-1*, *ProTT1:gTT16;tt16-1*, *ProTT1:gTT16-GUS* and *ProSTK:gSTK-GFP* lines were described previously (Mizzotti et al., 2014; Xu et al., 2016).

### Transgenic plants

The *Agrobacterium tumefaciens* strain C58C1 was used to stably transform *Arabidopsis* plants through the floral dip method (Clough and Bent, 1998). Transformants were selected by the appropriate hygromycin resistance and then checked by PCR assays. More than 20 independent transgenic lines were tested.

### Modified pseudo-Schiff propidium iodide (mPS-PI) staining

This protocol allows staining of cell walls of fixed plant material (Xu et al., 2016). More than 30 independent seeds or ovules were analyzed for each genotype and time point.

### Microscopy

mPS-PI and GFP fluorescent imaging was conducted with a Leica TCS-SP5 spectral confocal laser scanning microscope. Electron microscopy analyses were conducted as previously described (Xu et al., 2016).

### Quantitative morphological analyses

The central longitudinal section of seeds at 4 DAF, imaged using mPS-PI, was segmented into individual cells using CellSeT software (Pound et al., 2012). The length of the entire endothelium tissue and of individual ii1′ cells along the proximal-distal axis were calculated using ImageJ (Schneider et al., 2012). Area and perimeter of ii1′ cells were calculated with CellSeT. Cell roundness was determined for each ii1′ cell as:


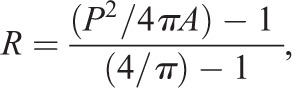


where *R*, *P* and *A* represent roundness, perimeter and area of the cell, respectively. Length and distance from the first proximal endothelium cell were used to determine the position of each ii1′ cell along the proximal-distal axis. Since seeds display a degree of variability in length and cell number across individuals and genotypes, we arbitrarily sampled 201 points uniformly distributed along the proximal-distal axis of each seed coat analyzed. Point 0 was fixed at the proximal side of the first proximal endothelium cell, whereas point 201 was set at the distal side of the first endothelium cell reaching the embryo suspensor. For each point, we determined the area and roundness of the ii1′ cell encompassing it. We then calculated the average ii1′ cell roundness and area at each specific point. Owing to variability in ii1′ position along the proximal-distal axis in each genotype, the extreme proximal and distal points are represented by fewer cells than the points at the curving zone.
